# Surgery for CNS Tumors in the Brazilian National Health Care System

**DOI:** 10.1200/JGO.2016.004911

**Published:** 2016-07-06

**Authors:** Luciola Pontes, Maryam Nemati Shafaee, Benjamin Haaland, Gilberto Lopes

**Affiliations:** **Luciola Pontes**, Hospital das Clínicas Instituto do Coração, São Paulo, Brazil; **Maryam Nemati Shafaee**, MD Anderson Cancer Center, Houston, TX; **Benjamin Haaland**, Georgia Institute of Technology, Atlanta, GA; and **Gilberto Lopes**, Oncoclinicas Group, São Paulo, Brazil, and The Johns Hopkins University, Baltimore, MD.

## Abstract

**Purpose:**

Resource limitations in low- and middle-income countries make the management of CNS tumors challenging, particularly in Brazil, a country with major disparities in socioeconomic status and access to health care. We aimed to evaluate cancer-related neurosurgical procedures in the public health care system.

**Methods:**

On the basis of Brazil’s public health system database, we collected data for neurosurgical procedures related to CNS tumors performed between January 2008 and November 2013. Information about the number of procedures, costs, length of stay, and number of inpatient deaths were analyzed for each state and then correlated to the state-specific population, gross domestic product per capita, and number of procedures.

**Results:**

In all, 57,361 procedures were performed, the majority of them in the Southeast region. The mean length of hospital stay was 14.4 days, but longer hospital stay was reported for patients treated in the North. The inpatient mortality rate was 7.11%. Mortality rates decreased as the number of procedures (*P* < .001), gross domestic product per capita (*P* < .001), or state population increased (*P* < .001). On multivariate analysis, only the number of procedures (odds ratio, 0.93; 95% CI, 0.91 to 0.96; *P* < .001) and state population (odds ratio, 1.25; 95% CI, 1.13 to 1.38; *P* < .001) had an independent association with mortality.

**Conclusion:**

To the best of our knowledge, this is the first study to evaluate disparities in CNS tumor surgery in a middle-income country, confirming that regional disparities exist and that clinical and economic outcomes correlate with income level, number of procedures, and state population.

## INTRODUCTION

Brain malignancies are among the three highest-cost cancers in the United States.^1^ The mainstay of therapy for the majority of CNS tumors is surgery, which is often followed by radiation therapy.^2^ Many factors have been reported to affect the outcome of surgical management for CNS tumors. These factors generally fall under the two broad categories of patient-related factors and hospital-related factors. More favorable outcomes have been reported in patients with active health insurance status^3^ and fewer comorbid conditions.^4^ Hospital-related positive predictors of improved outcomes include a hospital infrastructure geared towards high-volume neurosurgical procedures^5^ and high socioeconomic status of the patient population served by the hospital.^6^ In addition, racial and ethnic disparities in access to high-volume neuro-oncologic care and final health outcomes have been reported to disproportionately affect the African American and Hispanic patient population in the United States.^7^ African American patients are reported to present with more advanced disease, undergo neurosurgical procedures in low-volume centers, experience more procedure-related mortality, and experience poorer discharge conditions after craniotomy compared with white patients.^8^ Geographic maldistribution of oncologic specialty care centers has also been associated with unfavorable outcomes, and addressing the variation in regional resources has been suggested as a way to narrow the disparity gap in neuro-oncologic care in the United States.^6^

On a global scale, in low- and middle-income countries where availability of resources, or lack thereof, directly dictates lower investment on diagnostics and treatments,^9^ management of CNS tumors may pose different challenges. The annual incidence rate of primary CNS tumors globally is reported to be higher for males compared with females (3.7 *v* 2.6 per 100,000 person-years) and higher in developed countries compared with developing countries.^10^ According to a recent report on global status of cancer in 2013 by the Institute for Health Metrics and Evaluation, 3.6% of cancers in developing countries are primary tumors of the CNS.^11^ There is no uniform approach to how different developing countries allocate resources for management of primary CNS tumors. In this article, we aim to provide a country-wide picture of the state of surgical management for primary brain tumors in Brazil. The fifth largest and fifth most populous country in the world, Brazil is riddled with socioeconomic inequalities, which consequently translates into palpable disparities across all aspects of cancer care.^12^ The country is geopolitically subdivided into five regions: North, Northeast, South, Southeast, and Midwest. Each of these regions is investigated separately for frequency of neurosurgical operations for CNS tumors. Here we attempt to identify opportunities for developing cost-effective approaches to address the burden of primary CNS tumors and improve their surgical management.

## METHODS

The Brazilian public health system database (DATASUS) was reviewed for data collected between January 2008 and November 2013. All neurosurgical procedures related to primary CNS tumors were identified. Detailed information, including number of procedures, costs of each procedure and cost as a whole, length of inpatient hospital stay, and incidence of inpatient mortality were extracted for each state, and then they were associated with state-specific population, gross domestic product (GDP) per capita, and number of procedures.

Relationships among state-specific potential predictive variables such as population, number of procedures, and GDP per capita were summarized in terms of Spearman’s rank correlation. The relationships between state-specific respective outcomes, mortality, cost, length of inpatient hospitalization, and predictive variables were assessed in the context of univariate and multivariate generalized linear models, with a logit link for mortality, identity links for cost and length of hospitalization, and state-specific observations weighted by number of procedures.

## RESULTS

Between January 2008 and November 2013, a total of 57,361 procedures pertaining to management of primary CNS tumors were identified on retrospective review of procedures logged in Brazil’s public health system records. Regional population was strongly associated with number of procedures (Spearman’s rank correlation, 0.91; *P* < .001). There was no strong evidence that either population or number of procedures was related to GDP per capita (Spearman’s rank correlations were 0.17 [*P* = .404] and 0.33 [*P* = .090], respectively).

**Fig 1:**
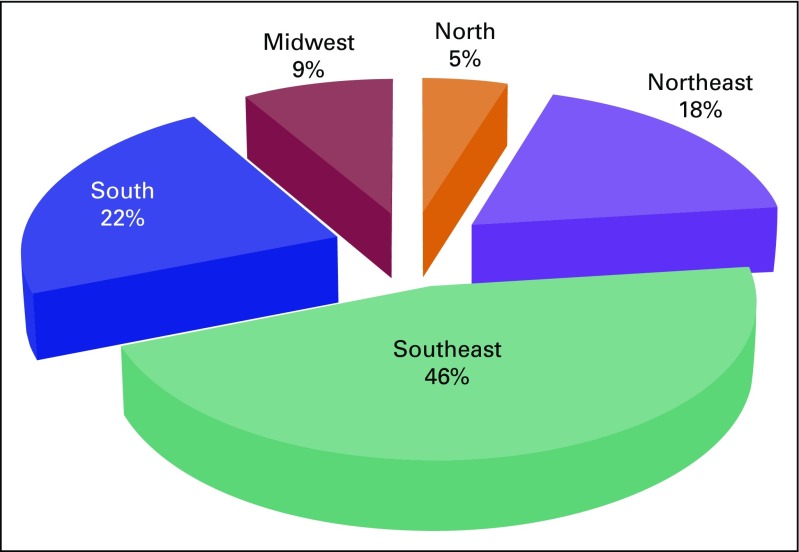
Distribution of neurosurgical procedures according to region. Used with permission. © 2014 American Society of Clinical Oncology. All rights reserved.

The highest number of neurosurgical procedures (46%) was performed in the Southeast region, followed by 22% in the South, 18% in the Northeast, 9% in the Midwest, and 5% in the North (Fig 1). The mean length of inpatient hospital stay was 14.4 days (95% CI, 13.1 to 15.7 days).

**Fig 2:**
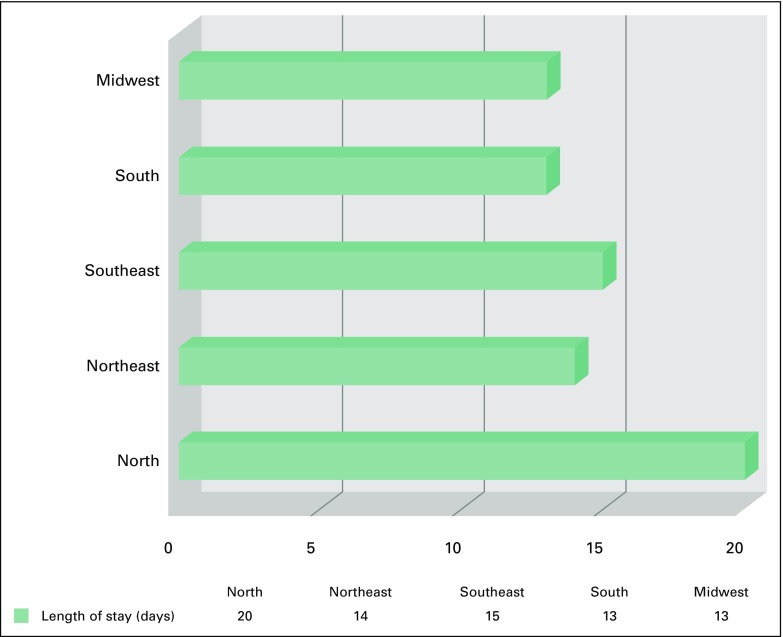
Length of hospital stay. Used with permission. © 2014 American Society of Clinical Oncology. All rights reserved.

The duration of hospitalization was significantly longer for patients treated in the North region (20 days) and shortest in the South and Midwest regions (13 days), as seen in Figure 2. On univariate analysis, there was no association between the number of days of hospitalization and number of procedures, GDP per capita, or regional population. On multivariate analysis, number of procedures, GDP per capita, and the regional population each had an independent association with number of days of hospitalization. For fixed GDP per capita and population, days of hospitalization tended to decrease as number of procedures increased. For a fixed number of procedures and population, days of hospitalization tended to increase as GDP per capita increased, and for a fixed number of procedures and GDP per capita, days of hospitalization tended to increase as population increased.

**Fig 3:**
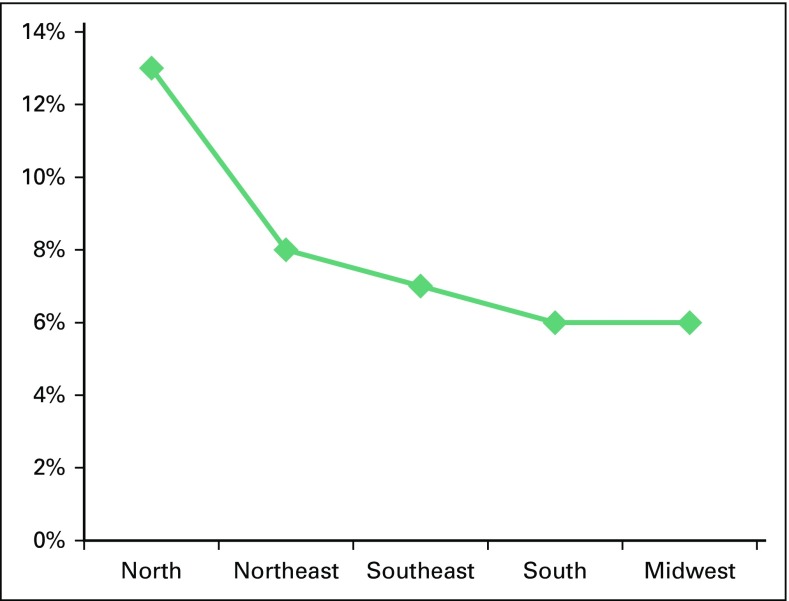
Mortality rate according to region. Used with permission. © 2014 American Society of Clinical Oncology. All rights reserved.

A total of 4,079 inpatient deaths were reported, translating into an inpatient mortality rate of 7.11%. Highest rates were seen in the North (13%). South and Midwest regions had the lowest procedure related mortality rate, 6% each (Fig 3). On univariate analysis, an inverse relationship was found between the mortality rates and number of procedures (*P* < .001), GDP per capita (*P* < .001), and state population (*P* < .001). On multivariate analysis, number of procedures (odds ratio [OR], 0.93; 95% CI, 0.91 to 0.96; *P* < .001) and population (OR, 1.25; 95% CI, 1.13 to 1.38; *P* < .001) were found to have an independent association with inpatient mortality.

**Fig 4:**
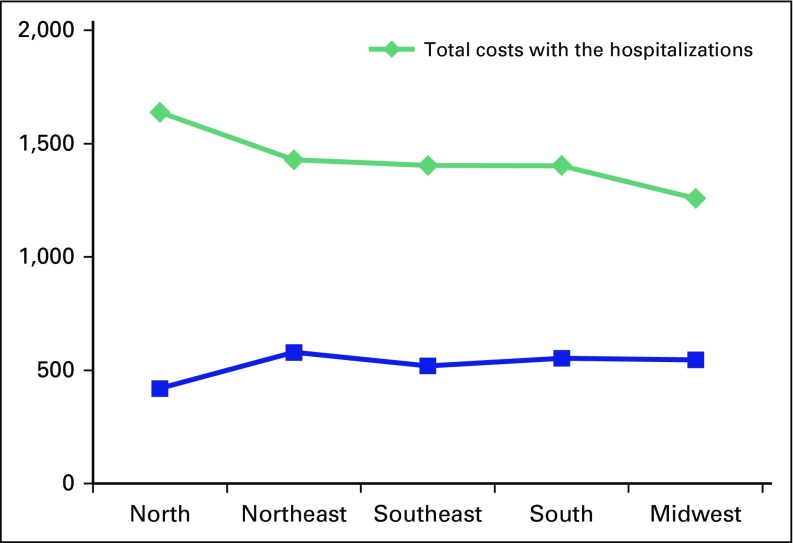
Cost per admission according to region. Used with permission. © 2014 American Society of Clinical Oncology. All rights reserved.

Total cost for the 57,361 procedures performed was calculated at US$108,363,802. Average cost per admission was US$1,889. For this calculation, the currency rate of 1 US$ = 2.40 Brazilian Real (R$) was used. The rate stands at 1 US$ = 4 R$ at this time. For a fixed number of procedures and population, the average cost per state tended to decrease as GDP per capita increased (US$250 decrease per US$10,000 per capita GDP; 95% CI, US$100 to US$400; *P* = .001). The North region was again found to stand out as having the highest cost per hospitalization and the lowest number of reimbursements to health care professionals per hospitalization event (Fig 4).

## DISCUSSION

Disparities in cancer care are a matter of public health concern worldwide. Socioeconomic disparities exist not only in different regions of the globe, but also in different regions of a single country.^13^ This means that when devising health policies, resource allocation needs to be made with areas of highest disparity in mind. This signifies the importance of careful assessment of the magnitude of disparities and their determinants before investing in remedial work.

In the United States, racial disparities in disease outcomes, not immediately explainable by differences in disease biology, are well documented. Race is noted to represent a crude measure of many other factors influencing the risks and treatment of disease. The US Department of Health and Human Services has officially acknowledged these disparities as well as actions taken by the government agencies in addressing them.^14^

Our study attempted to identify areas of disparity with regard to surgical management of CNS tumors in the middle-income country of Brazil. We found significant differences in the frequency of procedures and inpatient mortality rates between different regions. Duration of inpatient stay and mortality rates were highest in the North. This region was also significant for having the lowest number of procedures compared with the other four regions. The North is notable for having the largest land mass and lowest GDP of the five regions. Demographically, this area is home to only 6% of the country’s population, which includes the largest community of Native Amerindians. The lower GDP, higher land mass, and smaller population density in this region compared with the other regions in the country could influence the lower rates of surgical management of CNS tumors through potential lack of access, lower number of hospitals per population, greater distances to travel, fewer health care professionals, and fewer training facilities.

The Southeast region, which is home to 38% of the population and which has the highest GDP per capita, predictably experiences the highest number of neurosurgical procedures. The other three regions of the country fall on a spectrum with little variability in terms of hospital stay, mortality rates, and cost.

Average inpatient mortality rate postsurgery for CNS tumors in the United States is reported to be between 1.28% and 2.8%, depending on the insurance status of the patient.^3^ The rate in Brazil is considerably higher, at an average of 7.11%, with wide variation in the five different geopolitical areas.

Further studies are warranted to elucidate the causes of the high mortality from primary CNS tumors seen in Brazil as a whole. An area of future endeavor could be evaluating access to radiation therapy after surgical resection of CNS tumors.

In summary, this is the first study, to the best of our knowledge, to evaluate disparities in CNS tumor surgery in a middle-income country, confirming that regional disparities exist within a country under single governance. This study confirms that clinical and economic outcomes correlate with income level, number of procedures, and regional population.

## References

[1] Chang S, Long SR, Kutikova L (2004). Estimating the cost of cancer: Results on the basis of claims data analyses for cancer patients diagnosed with seven types of cancer during 1999 to 2000. J Clin Oncol.

[2] Campos S, Davey P, Hird A (2009). Brain metastasis from an unknown primary, or primary brain tumour? A diagnostic dilemma. Curr Oncol.

[3] Momin EN, Adams H, Shinohara RT (2012). Postoperative mortality after surgery for brain tumors by patient insurance status in the United States. Arch Surg.

[4] Hooten KG, Neal D, Lovaton Espadin RE (2015). Insurance status influences the rates of reportable quality metrics in brain tumor patients: A nationwide inpatient sample study. Neurosurgery.

[5] Trinh VT, Davies JM, Berger MS (2015). Surgery for primary supratentorial brain tumors in the United States, 2000-2009: Effect of provider and hospital caseload on complication rates. J Neurosurg.

[6] Aneja S, Khullar D, Yu JB (2013). The influence of regional health system characteristics on the surgical management and receipt of post operative radiation therapy for glioblastoma multiforme. J Neurooncol.

[7] Mukherjee D, Zaidi HA, Kosztowski T (2010). Disparities in access to neuro-oncologic care in the United States. Arch Surg.

[8] Curry WT Jr, Carter BS, Barker FG II: Racial, ethnic, and socioeconomic disparities in patient outcomes after craniotomy for tumor in adult patients in the United States, 1988-2004. Neurosurgery 66:427-437, 2010; discussion 437-438 10.1227/01.NEU.0000365265.10141.8E 20124933

[9] Huerta E, Grey N (2007). Cancer control opportunities in low- and middle-income countries. CA Cancer J Clin.

[10] Bondy ML, Scheurer ME, Malmer B (2008). Brain tumor epidemiology: Consensus from the Brain Tumor Epidemiology Consortium. Cancer.

[11] Fitzmaurice C, Dicker D, Pain A (2015). The global burden of cancer 2013. JAMA Oncol.

[12] de Souza JA, Hunt B, Asirwa FC, et al: Global health equity: Cancer care outcome disparities in high-, middle-, and low-income countries. J Clin Oncol 34:6-13, 2016 10.1200/JCO.2015.62.2860 PMC5795715 26578608

[13] Olopade OI, Schwartsmann G, Saijo N (2006). Disparities in cancer care: A worldwide perspective and roadmap for change. J Clin Oncol.

[14] Sampson JH, Bagley CA, Carson BS (2012). Disparities in care. J Neurosurg.

